# Therapeutic Challenges in Metastatic Castration-Resistant Prostate Cancer: A Case Study and Review of Prostate-Specific Membrane Antigen-Targeted Therapy Failures in Highly Prostate-Specific Membrane Antigen-Avid Disease

**DOI:** 10.7759/cureus.76224

**Published:** 2024-12-22

**Authors:** Serin Moghrabi, Raghad Al-Houwari, Saad Ruzzeh, Akram Al-Ibraheem

**Affiliations:** 1 Nuclear Medicine, King Hussein Cancer Center, Amman, JOR

**Keywords:** 177lu-psma, 225ac-psma, highly psma-avid, mcrpc, therapy failure

## Abstract

Prostate cancer (PCa) is a leading cause of cancer-related deaths globally, with metastatic castration-resistant prostate cancer (mCRPC) posing significant treatment challenges. This case report discusses a 65-year-old male with mCRPC who initially responded to hormonal therapy but later showed disease progression despite additional chemotherapy. He subsequently received ^177^Lu-PSMA and ^225^Ac-PSMA therapies, both of which failed to halt disease progression despite having an intense PSMA avid metastatic disease. This case highlights the complexity of treatment responses in mCRPC, particularly when intense PSMA avidity does not correlate with therapeutic effectiveness. It suggests that underlying genomic alterations may contribute to resistance, highlighting the potential role of circulating tumor DNA (ctDNA) in identifying biomarkers that predict treatment outcomes. Further research is necessary to explore these genomics, along with other potential resistance mechanisms, to enhance personalized treatment strategies for patients with mCRPC.

## Introduction

Prostate cancer (PCa) is a major health issue, ranking as the second most prevalent cancer and the fifth leading cause of cancer-related deaths among men globally [1]. The progression to metastatic castration-resistant prostate cancer (mCRPC) presents significant challenges due to resistance to hormonal therapies and rapid disease progression [2]. Prostate-specific membrane antigen (PSMA)-targeted therapies, such as 177Lu-PSMA and 225Ac-PSMA, have emerged as promising treatment options. 177Lu-PSMA targets PSMA, a protein highly expressed in PCa cells, leading to improved patient outcomes with a prostate-specific antigen (PSA) response rate of 60-70% and a median progression-free survival of about eight months [3-5]. In addition, 225Ac-PSMA therapy, using alpha particle emissions, is showing promise for patients resistant to 177Lu-PSMA, offering enhanced tumor destruction [6].

The heterogeneity of response to PSMA radioligand therapy (PSMA PRLT) in mCRPC is affected by various factors like baseline PSMA expression and tumor volume, which are assessed by 68Ga-PSMA positron emission tomography/computed tomography (PET/CT) [7]. Higher PSMA intensity predicts better therapy response, while larger tumor volumes indicate poorer outcomes [8]. In addition, a dual PET/CT approach can be useful to identify tumoral heterogeneity through the use of fluorine-18 fluorodeoxyglucose 18F-FDG PET/CT and 68Ga-PSMA PET/CT. 18F-FDG-avid mismatch lesions, with low PSMA expression, are associated with worse outcomes [9]. Moreover, previous taxane chemotherapy reduces treatment efficacy and overall survival [10]. These factors can impact the delivery and uptake of the radiolabeled compound, ultimately affecting the therapeutic response.

This case report highlights a scenario where a patient did not respond to PSMA PRLT despite intense 68Ga-PSMA avidity in the metastatic lesions, adequate localization of the therapeutic radiotracer in the known metastatic lesions, and no mismatched FDG-avid lesions, contrary to the generally known positive correlation between high PSMA expression and favorable response to PSMA PRLT in mCRPC.

This lack of responsiveness may be attributed to underlying genomic alterations, particularly through the evaluation of circulating tumor DNA (ctDNA), which can predict treatment resistance. A decline in ctDNA levels during early cycles of 177Lu-PSMA therapy has been associated with positive treatment responses, as it reflects reduced tumor burden. Patients with significant ctDNA reduction are more likely to see corresponding decreases in PSA levels, highlighting the potential of ctDNA as a predictive biomarker for personalized treatment strategies [11,12]. However, despite these promising insights, the direct correlation between specific genomic changes and PSMA PRLT non-responsiveness requires further validation through extensive research.

## Case presentation

A 65-year-old male presented in February 2022 with complaints of lower abdominal pain and lower urinary tract symptoms, like urgency, dribbling, and a sensation of incomplete bladder emptying. The severity of the urgency was moderate, occurring multiple times during the day and night, while the dribbling was noticeable after urination, significantly affecting his daily activities. Physical examination revealed a hard prostate with a nodular contour during a digital rectal exam (DRE), with no tenderness or asymmetry noted. Laboratory tests were unremarkable, except for a significantly elevated prostate-specific antigen (PSA) level of 299 ng/ml. Consequently, a prostate transrectal ultrasound (TRUS) biopsy was performed, revealing prostatic acinar adenocarcinoma involving both prostate lobes, with a Gleason score of seven. His initial 68Ga-PSMA PET/CT scan confirmed the presence of PSMA avid multifocal prostate gland lesions associated with PSMA avid metastatic abdominopelvic and mediastinal lymphadenopathy, as well as multiple metastatic bone lesions, resulting in a staging of T3bN1M1c, with the most prominent lesion located in the right acetabulum bone, with a maximum standard uptake value (SUV_max_) of 37.0.

The initial treatment plan involved hormonal therapy with abiraterone, to which the patient initially responded well, as evidenced by a reduction in PSA levels to 14 ng/ml dated in December 2022. However, this favorable response was short-lived, as the patient's PSA level began to rise again until it reached 71 ng/ml in March 2023. This rise in PSA, despite ongoing hormonal therapy, marked the time point at which the clinical diagnosis of mCRPC was affirmed. The 68Ga-PSMA PET/CT scan back then showed newly seen metastatic PSMA avid bone lesions involving the skull, right scapula, and left iliac bone, along with a more prominent appearance of the known right acetabular lesion with an SUV_max_ of 95.1 and total lesion PSMA (TL-PSMA) of 918.7; however, the scan showed regression in the mediastinal and abdominopelvic lymph nodes. This necessitated the multidisciplinary team (MDT) to add chemotherapy as the new line of treatment.

**Figure 1:**
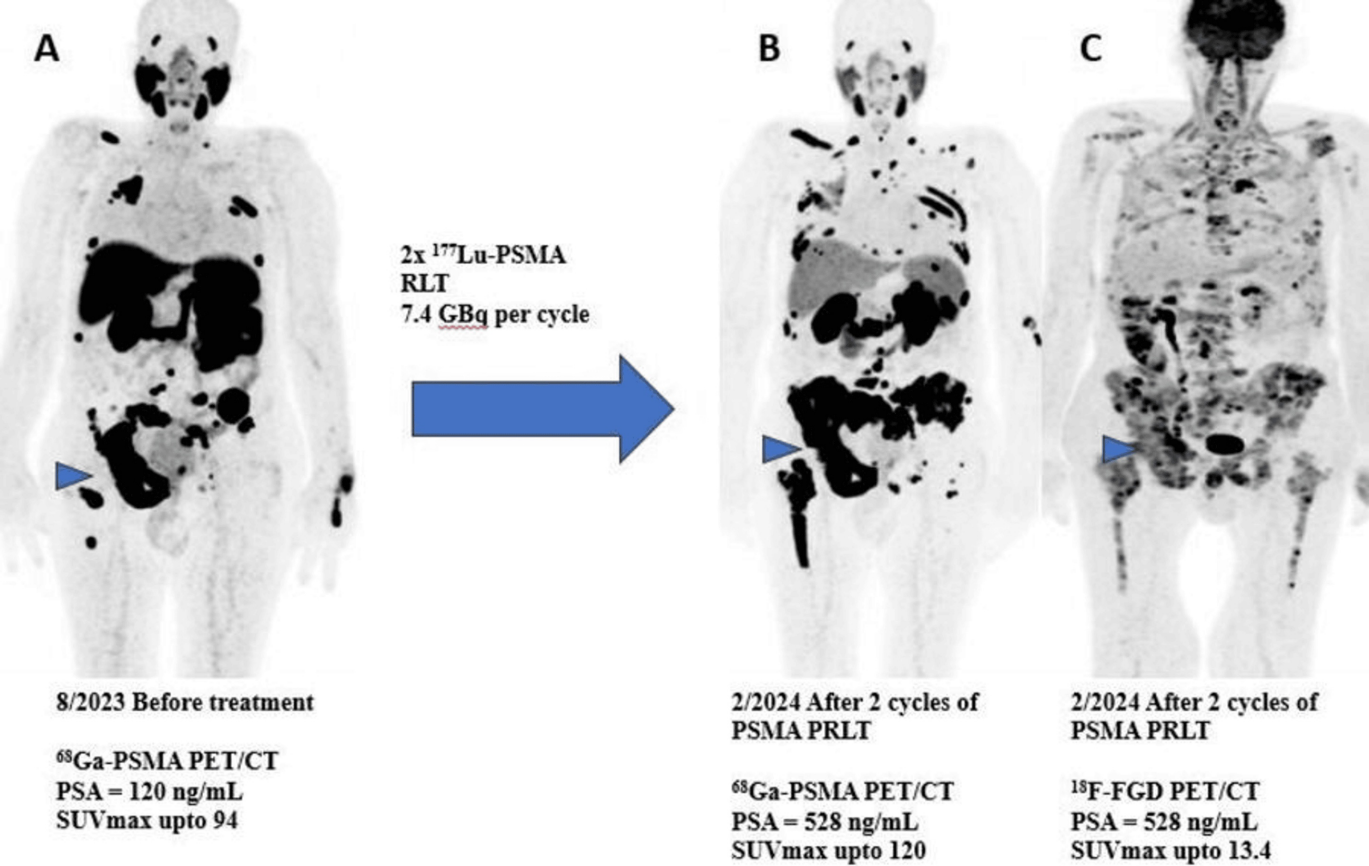
Imaging results before and after prostate-specific membrane antigen radioligand therapy (PSMA PRLT). Imaging results before and after prostate-specific membrane antigen radioligand therapy (PSMA PRLT) in a patient with metastatic castration-resistant prostate cancer (mCRPC). (A) Pre-treatment 68Ga-PSMA PET/CT (August 2023) shows intense PSMA avidity in the primary prostate gland tumor, lymph nodes, and bone metastases. The arrowhead indicates the right acetabular lesion, with an SUVmax of 94. The patient’s PSA level is 120 ng/mL before treatment. (B) After two cycles of 177Lu-PSMA therapy (February 2024), the post-treatment 68Ga-PSMA PET/CT shows progression of bone metastases, with an SUV_max_ of 120. The right acetabular lesion (indicated by the arrowhead) continues to demonstrate high PSMA avidity. PSA level increased to 528 ng/mL. (C) Post-treatment 18F-FDG PET/CT shows lower and heterogeneous FDG uptake in the same bone metastases, with an SUV_max_ of 13.4, indicating a shift in metabolic activity compared to PSMA avidity. The PSA level remains elevated at 528 ng/mL.

Despite the patient receiving three cycles of docetaxel, his condition continued to deteriorate, revealed by a new 68Ga-PSMA PET/CT scan performed in August 2023, which confirmed disease progression, in terms of interval development of intense PSMA avid bone lesions in the rib cage, spine, bilateral pelvic bones, and right femur, along with more prominent appearance of the known metastatic bone lesions with the SUV_max_ reaching 94.0, SUV_mean_ = 52.4, and TL-PSMA = 2376 in the right acetabulum bone (Figure 1), with the PSA level at this stage reaching 120 ng/ml. However, there was complete resolution of the known mediastinal lymphadenopathy, with further regression of the pelvic lymph nodes.

**Figure 2:**
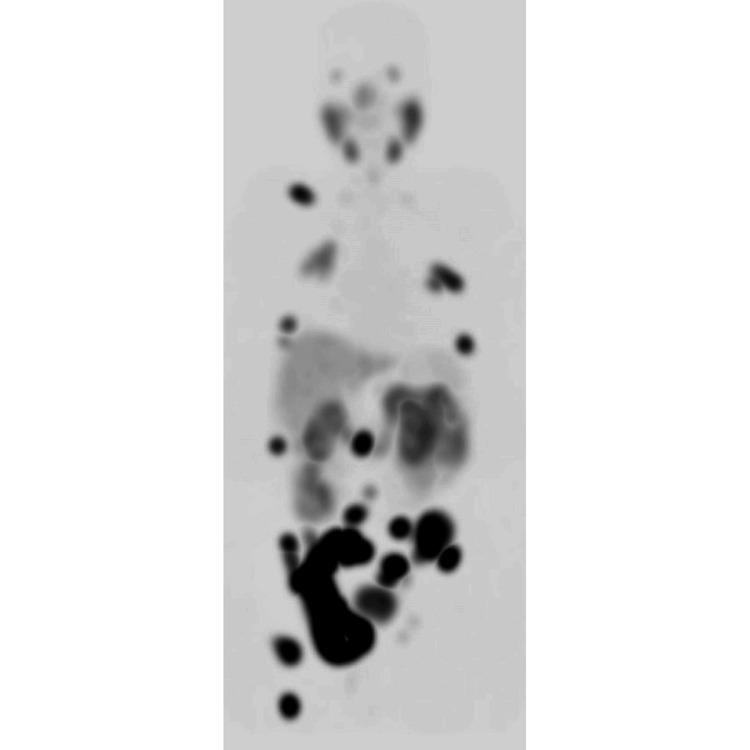
Maximum intensity projection of the second 177Lu-PSMA SPECT/CT scan. Maximum intensity projection of the second 177Lu-PSMA single-photon emission computed tomography/computed tomography (SPECT/CT) scan shows adequate localization of the radiotracer within the known metastases.

The MDT decided afterward to refer him to the nuclear medicine clinic for possible PSMA PRLT. After thorough evaluation and eligibility assessment, the patient was given the first dose of 7.4 Gigabecquerel (GBq) of 177Lu-PSMA therapy in October 2023, and the following single-photon emission computed tomography/computed tomography (SPECT/CT) scan showed adequate localization of the therapeutic radiotracer in the known metastatic lesions, so the second dose of 7.4 GBq of 177Lu-PSMA therapy was given in December 2023. Moreover, the following SPECT/CT scan showed similar adequate radiotracer localization, anticipating tumor activity in response to PSMA PRLT (Figure 2). The patient reported stable overall health with minimal symptoms following treatment, including nausea and fatigue, graded as 1 according to the Common Terminology Criteria for Adverse Events (CTCAE) version 5. However, based on the radiological and biochemical workup done after the second dose in January 2024, the patient was considered not responding to 177Lu-PSMA therapy since his general health deteriorated, his PSA level kept on rising reaching 528 ng/ml, and this follow-up 68Ga-PSMA PET/CT scan showed predominant disease progression in the known metastatic bone lesions as the previous right acetabular bone lesions currently involving the whole right hemipelvis, with relatively stable appearance of the known metastatic pelvic lymphadenopathy (Figure 1). An 18F-FGD PET/CT scan was ordered for the evaluation of possible dedifferentiation, it showed heterogeneous FDG uptake distribution in the known bone metastases with a heterogenous but much less intense pattern when compared to PSMA expression. For instance, the right acetabular lesion has an SUV_max _of 13.4 on the FDG scan with SUV_mean_ = 6.5; however, the pelvic lymph nodes showed comparable FDG uptake to the previous PSMA uptake (Figure 1). Thus, the decision was to switch to a more potent PSMA PRLT, so the patient was given 4 MBq of 225Ac-PSMA in March 2024. However, the patient’s condition got worse as he complained of severe progressive back pain and xerostomia, graded as 3 according to the CTCAE version 5, and the PSA level reached 1569 ng/ml a month later, resulting in the discontinuation of PSMA PRLT and referring him to the palliative clinic.

## Discussion

The variability in PSMA PRLT outcomes among mCRPC patients, as demonstrated in this case report, underscores the complexity of treatment response. While high baseline PSMA expression and lower tumor volumes typically predict better therapeutic responses [8], individual outcomes can deviate significantly from these trends. This case, where the patient showed intense 68Ga-PSMA avidity but did not respond to PSMA PRLT, underscores the limitations of current predictive markers and the need to refine these criteria.

PSMA PRLT's effectiveness in treating mCRPC varies among patients due to several influencing factors. To predict the efficacy of PSMA PRLT, it is crucial to understand how baseline metabolism and PSMA expression interact, as indicated by 68Ga-PSMA PET/CT imaging [13]. As Hofman et al. showed in the phase 2 TheraP trial, a higher SUV_mean_ on baseline 68Ga-PSMA PET/CT was associated with a favorable response to 177Lu-PSMA therapy compared to cabazitaxel in mCRPC patients [7]. This suggests that the intensity of PSMA expression is a potent biomarker, guiding clinicians in identifying those individuals most likely to benefit from this targeted radionuclide therapy [7]. The tumor volume, as assessed by 68Ga-PSMA PET/CT imaging, plays a significant role in predicting the response to 177Lu-PSMA therapy in patients with mCRPC [7]. A higher baseline tumor volume, characterized by a greater number of PSMA-avid lesions, is associated with a poorer response to treatment [8]. Patients with a high tumor burden (>20 lesions) on baseline 68Ga-PSMA PET/CT exhibit significantly elevated PSA concentrations before treatment, suggesting a direct correlation between the extent of disease and the overall burden of PCa cells secreting PSA [8].

Previous chemotherapy appears to have a negative impact on overall survival (OS) in patients receiving 177Lu-PSMA therapy for mCRPC [14]. Patients who have undergone prior chemotherapeutic regimens, such as docetaxel or cabazitaxel, often present with more aggressive and resistant tumor phenotypes, which can adversely affect the efficacy of subsequent 177Lu-PSMA treatment [14]. A recent systematic review and meta-analysis evaluated the impact of prior taxane chemotherapy on the efficacy of 177Lu-PSMA radioligand therapy in patients with mCRPC [9]. The key findings indicate that taxane-naïve patients had significantly better odds of achieving a biochemical response (PSA reduction) after 177Lu-PSMA compared to those who received prior taxane chemotherapy. An individual patient data meta-analysis revealed a higher PSA response rate, significantly better PFS, and OS in taxane-naïve patients versus taxane-treated patients [10].

The standardized uptake value mean (SUV_mean_) plays a pivotal role in predicting the response to 177Lu-PSMA therapy in patients with mCRPC [15]. SUV_mean_, a semiquantitative PET parameter, reflects the average tracer uptake in PSMA-expressing tumor cells and is an indicator of their metabolic activity [16]. Studies have consistently shown that patients with a higher baseline SUV_mean_ in PSMA-PET/CT imaging tend to respond better to 177Lu-PSMA therapy, likely due to increased PSMA expression in more metabolically active tumor regions, allowing for more efficient radioligand binding and targeted radiation [15]. Although a precise SUV_mean _cutoff varies, patients with values above 10 typically have better outcomes in terms of progression-free survival (PFS) and (OS)[15] . However, our patient, despite having a high SUV_mean_ of 52.4, did not exhibit a favorable response to therapy, highlighting that SUV_mean_ alone may not always predict therapeutic success.

18F-FDG PET/CT scan plays a role in evaluating dedifferentiation in mCRPC by identifying FDG-avid lesions that exhibit low or no PSMA expression, known as “mismatch lesions” [17]. These lesions are associated with a poor response to 177Lu-PSMA therapy, indicating a more aggressive disease course and reduced OS rates [17]. The presence of FDG-avid lesions, characterized by increased glucose metabolism and low PSMA expression, suggests that these tumors may not be as responsive to PSMA PRLT [18]. However, the role of 18F-FDG PET in evaluating patients before the start of PSMA PRLT is not yet well-established [19]. Findings from a recent study conducted by Seifert et al. revealed that only 3% of patients were deemed ineligible for PSMA PRLT based on 18F-FDG/PSMA mismatch findings on 18F-FDG and PSMA PET scans [19]. Based on these new recommendations, a baseline FDG PET/CT scan was not performed for our patient before proceeding with PSMA PRLT.

In addition, the metabolic tumor volume (MTV) derived from FDG PET/CT scans is a significant prognostic biomarker in mCRPC, as it reflects the total volume of metabolically active tumor burden [15]. Larger MTV values, typically over 200 mL, are associated with worse outcomes and a higher likelihood of disease progression [15]. In our patient’s case, although the MTV was relatively low at 45.4 mL, which is below the 200 mL threshold, the response to therapy was still poor. This suggests that while MTV can provide valuable information on tumor burden, it is not always a definitive predictor of therapeutic response, and additional factors may contribute to treatment resistance.

Our patient, a 65-year-old man with PCa, initially presented with intense, homogeneous PSMA-avid lesions on 68Ga-PSMA PET/CT, including a right acetabular bone lesion with an SUV_max_ of 37. Despite hormonal therapy and chemotherapy, his disease progressed, leading to 177Lu-PSMA radioligand therapy. Initial 177Lu-PSMA SPECT/CT scans showed adequate localization of bone lesions, but PSA levels continued to rise. Subsequent 68Ga-PSMA PET/CT revealed frank progression, and 18F-FDG PET/CT showed heterogeneous FDG uptake in the bone metastases, less intense than PSMA expression. Rising PSA and imaging findings confirmed unresponsiveness to 177Lu-PSMA and 225Ac-PSMA, with disease progression despite high PSMA expression.

The lack of response to PSMA PRLT in patients exhibiting intense PSMA avidity and low FDG avidity in known metastases may be attributed to underlying genomic factors. Recent studies have demonstrated that the evaluation of ctDNA can identify potential biomarkers, thereby aiding in the prediction of PSMA PRLT response [11,12]. The reduction in ctDNA levels from baseline to the early cycles of 177Lu-PSMA therapy has been closely associated with positive treatment outcomes. This decline in ctDNA levels serves as an early indicator of therapeutic efficacy, reflecting a decrease in the tumor burden as the therapy effectively targets and eliminates PSMA-expressing cancer cells. Patients who exhibit a significant reduction in ctDNA during early treatment cycles are more likely to experience a corresponding decrease in PSA levels [12]. These findings underline the potential of ctDNA as a predictive biomarker for assessing which patients may respond best to 177Lu-PSMA therapy, allowing for more tailored treatment approaches.

A few studies examined the genomic alterations in two key pathways: the androgen receptor (AR) and phosphatidylinositol-3-kinase (PI3K) signaling pathways [11,12]. These pathways play critical roles in the development and progression of PCa, and alterations can significantly impact therapy response [11,12]. The AR pathway regulates androgen receptor signaling, which is crucial for PCa growth. Mutations or amplifications in the AR pathway can increase AR activity and lead to resistance to androgen deprivation therapy (ADT), a standard PCa treatment [12]. Patients with AR pathway alterations were more likely to respond poorly to 177Lu-PSMA therapy [11, 12].

Similarly, the PI3K pathway, which is involved in cell survival, proliferation, and metabolism, can also affect therapy outcomes [11,12]. Mutations or amplifications in the PI3K pathway can enhance PI3K activity and lead to treatment resistance. Patients with PI3K pathway alterations were likewise more likely to be poor responders to 177Lu-PSMA therapy [11,12]. Unfortunately, genomic testing for these alterations is not yet available at our center and was not offered to our patient.

Vanwelkenhuyzen et al. conducted the genomic profiling analysis on the pre-treatment ctDNA samples from each patient in their study, who were treated with various types of treatments including chemotherapy and AR deprivation regimens, as well as 177Lu-PSMA therapy [11]. The study found that patients with alterations in either the AR or PI3K pathway experienced poorer overall response rates (ORR), disease control rates (DCR), and PFS compared to those without these alterations [11]. Specifically, patients with these pathway alterations had lower overall response rates (26% vs. 65%), lower disease control rates (61% vs. 87%), and shorter progression-free survival (3.9 months vs. 7.1 months) [11].

To combat this, one article proposed the co-administration of 177Lu-PSMA with a PI3K pathway inhibitor and/or a more potent AR pathway inhibitor may represent a strategy to overcome 177Lu-PSMA resistance [12].

To date, there has been no study examining the non-response of 225Ac-PSMA therapy and its correlation with genomic testing.

## Conclusions

This case report highlights the complexities in predicting the response to PSMA PRLT in mCRPC. Despite high baseline PSMA expression, high SUV_mean_, adequate localization of 177Lu-PSMA therapy to known metastatic lesions, and the absence of 18F-FDG/PSMA mismatch lesions, the patient’s progression persisted. This lack of response underscores the limitations of current predictive markers, such as PSMA intensity, tumor volume, and SUV_mean_.

Genomic alterations, particularly those in the androgen receptor (AR) and PI3K pathways, are emerging as potential contributors to treatment resistance and may explain why patients with high PSMA expression may still not respond to therapy. Although ctDNA analysis was not performed in this case, its potential to predict non-responsiveness to PSMA PRLT warrants further investigation. The patient’s progression despite both 177Lu-PSMA and 225Ac-PSMA therapies highlights the need for a more personalized approach, incorporating genomic profiling to refine treatment strategies and overcome resistance.
